# A qualitative study to inform the development of a new quality of life measure for surgery for prolapse, incontinence and mesh complications

**DOI:** 10.3389/fgwh.2026.1643835

**Published:** 2026-02-12

**Authors:** Francesca Taylor-Phillips, Alicia O’Cathain, Janice Connell, Mollie Price, Cat Brooke, Swati Jha, Stergios Doumouchtsis, Thomas Gray, Stephen Radley, Victoria Fisher, Georgina Forshall, Georgina L. Jones

**Affiliations:** 1 Psychology, School of Humanities and Social Science, Leeds Beckett University, Leeds, United Kingdom; 2 Sheffield Centre of Health and Related Research, University of Sheffield, Sheffield, United Kingdom; 3 Department of Urogynaecology, Sheffield Teaching Hospitals NHS Foundation Trust, Sheffield, United Kingdom; 4 Department of Obstetrics and Gynaecology, Epsom and St. Helier University Hospitals NHS Trust, Epsom, United Kingdom; 5 University of East Anglia, Norwich, United Kingdom

**Keywords:** patient reported outcome measure, pelvic floor surgery, prolapse, quality of life, urinary incontinence, vaginal mesh implants

## Abstract

**Background:**

The National Institute of Health and Care Excellence in the United Kingdom have recommended the development of a patient-reported outcome measure (PROM) specific to surgery for pelvic organ prolapse (POP), stress urinary incontinence (SUI), and complications of pelvic mesh surgery (MC).

**Aims:**

To identify all aspects of quality of life (QoL) that may be impacted by surgery for POP, SUI and MC, to inform the development of a new PROM.

**Method:**

Thirty-one patients who had undergone surgery for POP, SUI and MC (ranging from <6 months to >5 years ago) were purposively recruited from various National Health Service (NHS) Trusts, as well as community support groups for the conditions. Semi-structured interviews were undertaken over the phone or via video-conferencing software, and a framework approach was employed to analyse the data. A Patient and Public Involvement group, comprising seven women who had undergone surgery for POP, SUI and/or MC, were consulted on various aspects of the study.

**Findings:**

We identified nine themes for potential inclusion in the PROM. The themes are short-term impacts of surgery; long-term impacts on pre-surgery symptoms; pain and/or discomfort; impact on daily activities; social and leisure activities; emotional wellbeing; sexual activity; personal relationships; and work/education. While some participants showed improvements in these areas following surgery, it was also evident that for some, issues persisted, worsened, or developed post-surgery. Those in the sample who had previous surgery involving mesh reported worse QoL outcomes overall.

**Conclusion:**

The impact of surgery for POP, SUI and MC on QoL is multidimensional and complex. It is recommended that a future PROM encompasses the potential for improvement of symptoms, the failure of surgery to improve symptoms, the development of new symptoms after surgery, and the consequential positive and negative impacts of surgery on activity, roles, psychological well-being and ultimately QoL, in both the short and long term.

## 1 Introduction

Pelvic floor disorders including pelvic organ prolapse (POP—downward descent of pelvic organs into vagina) and stress urinary incontinence (SUI—leakage of urine related to increases in intra-abdominal pressure) are common. Estimates suggest that POP can affect between 40%–60% of women (1) and SUI between 20%–50% of women (2). Pelvic floor disorders have been shown to have a profound negative effect on physical, sexual, interpersonal, social, and emotional health, and subsequently overall well-being and quality of life (QoL) (3).

Various surgical treatments are available to treat POP and SUI, including the use of non-absorbable polypropylene meshes, which is now unavailable in some countries (4). Non-mesh surgical interventions are also available, such as anterior and posterior vaginal wall repair (colporrhaphy), vaginal hysterectomy, vaginal closure (colpocleisis), autologous fascial slings, colposuspension, and urethral bulking agents (5). It is estimated that 10%–20% of women will undergo surgery for pelvic floor disorders in their lifetime (6, 7).

Whilst non-mesh surgical interventions are currently considered to be effective for many patients and can improve QoL (8, 9), synthetic mesh surgery was introduced in the 1990's because of the high failure rate of native tissue repairs (10). It is estimated that approximately 1 in 5 patients undergoing pelvic floor surgery for POP or SUI require further treatment (11). However, it has been suggested that the evidence that supported the introduction and popularisation of vaginal mesh surgery for POP was of poor quality and lacked vigilant, long-term monitoring (12). Complications after transvaginal mesh surgery for POP are estimated to be between 15%–25% (13). Subsequently, a significant number of patients have reported life changing complications associated with these pelvic mesh implants (12).

Whilst there was significantly more evidence for the safety and efficacy of vaginal mesh to treat SUI, these are also associated with mesh complications (MC), and their use has been paused, banned, or greatly restricted in some countries (12). Guidelines have been produced recently to improve practice (14). In recent years, a growing number of qualitative studies on the experiences of patients who have had mesh surgery for POP or SUI reported significant harm with a profound negative impact on both their physical and mental health (15–21). However, evidence reviews, which include both mesh and non-mesh interventions, report a better QoL overall post-surgery (8, 22). There is also evidence of patients experiencing both short and long term symptomatic and functional adverse events after pelvic floor disorder surgery (23). Additionally, the level and type of impact on QoL can differ according to whether a quantitative or qualitative approach to data collection and analysis is used (24).

Given the various and sometimes contradictory surgery outcomes indicated, it has been recommended that Patient Reported Outcome Measures (PROMS) are used more widely and routinely in the assessment of benefits and risks of surgical interventions (12). In response, the National Institute of Health and Care Excellence (NICE) in the United Kingdom has called for the development of a new validated PROM that is specific to surgery for POP, SUI and MC. The PROM aims to improve understanding of the effects of these types of surgery and support treatment decision-making (4).

PROMs currently exist for pelvic floor disorders (25), but none have been developed specifically to measure changes in QoL post-surgery. We were funded to conduct a series of studies (collectively known as APPRAISE) (26) resulting in the development of a PROM, using the Food and Drug Administration (FDA) guidance on developing PROMs (27). To ensure content validity of the PROM, the wider body of research included undertaking systematic reviews (28, 29), secondary data analysis of a subset of data generated in the NIHR PURSUE study (30), content analysis of an existing comprehensive pelvic floor PROM (ePAQ-PF) (31), and qualitative interviews with patients who have had surgery for POP, SUI and MC. We also carried out extensive stakeholder engagement activities, including engaging with the patient advocacy group Sling the Mesh, and a webinar in collaboration with a local inclusivity champion to promote the study, start conversations and gain a better understanding of how diversity (e.g., disability, social and cultural heritage and faith) may impact patient experiences of living with these pelvic floor conditions. In this article, we present the findings from the qualitative interviews, in which we explored the views and experiences of patients who have had surgery for POP, SUI or MC, with the aim of identifying all aspects of QoL that may be impacted by surgery.

## 2 Methods

### 2.1 Design

We undertook 31 semi-structured interviews between May and November 2023, with patients who had surgery for POP, SUI, or MC. Ethical approval for this study was granted by the Health Research Authority (323441) and Leeds Beckett University ethics committee.

### 2.2 Patient and public involvement (PPI)

Two women with lived experience of adverse outcomes following urogynaecological surgery were co-applicants on the APPRAISE study. A further five PPI members, who had all undergone surgery for either POP, SUI or MC, shaped the wider APPRAISE project throughout by providing feedback and comments on participant facing documents, coding frameworks, data analysis and draft PROMs. In this study, PPI members helped develop the topic guide for the interviews, and one participated in a pilot interview which resulted in changes to the topic guide and recruitment procedures.

### 2.3 Recruitment

We undertook purposive sampling to recruit patients from the three surgical groups (i.e., POP, SUI, MC), aiming for 10 from each group. We also aimed for diversity in terms of socio-demographic characteristics (i.e., age, ethnicity and socio-economic background), outcome of surgery (i.e., positive, negative, mixed), and timing of interview post-surgery so we could explore both short term (i.e., <6mth months) and long-term (i.e., > 6 months) outcomes. To be included in the study, participants had to: i) have had surgery for POP or SUI either with or without the use of mesh [M]), or experienced mesh complications resulting in surgery (MC); ii) have had surgery at least 6 weeks previously and no longer than 20 years previously, iii) be 16 years or older (although we acknowledged they would likely be older than this) and, iv) had not previously taken part in a qualitative study about experiences of having these conditions or surgeries.

We recruited participants to the study from two sources. First, from three NHS Trusts in geographically diverse parts of England. POP, SUI and MC surgeons, sometimes with research nurse support, identified potential participants who met the inclusion criteria from review of referral letters to clinics, patients notes and surgical databases indicating where patients had relevant previous surgical procedures. Purposive sampling was undertaken to try and include patients with protected characteristics who are under-represented in urogynaecology research including patients from black and world majority backgrounds and those with gender dysphoria. Patients identified as having had relevant surgical procedures were provided with information about the study and a ‘consent to contact’ form. Those who were interested and completed the form were then contacted by members of the research team to check their eligibility for the study and, if they met the criteria, they were recruited for interview.

Second, information about the study was shared by community organisations (i.e., on their social media platforms, newsletters, and mailing lists). These organisations included: Sling the Mesh; MASIC Foundation; Bladder and Bowel Community; Bristol Mesh Centre; Pelvic Obstetric and Gynaecological Physiotherapy; Mesh Rectopexy Support and Action Group; and Bladder Health UK. Recruitment was also attempted through community organisations for people from ethnic minority communities or with physical disabilities or learning disabilities to try to ensure representation from underserved groups, but this was unsuccessful. Recruitment took place between May and October 2023 and interviews took place between June and November 2023. All of those recruited who met the sampling criteria were interviewed. All participants gave written informed consent.

### 2.4 Data collection

One researcher (JC) undertook all interviews. The majority (*n* = 27) were conducted over the phone, and the remainder (*n* = 4) using online video conferencing software. The interviews lasted between 38 and 1 h 54 min, with an average time of 1 h 4 min. It was not uncommon that this interview was the first time some participants had been given the opportunity to talk about their experiences of their conditions, symptoms and surgeries. As such, many participants relished the opportunity to tell their stories and to be heard, and others stuck to the topic guide without much elaboration. This is reflected in the disparity in interview length.

The topic guide included questions about: the type of surgery; what problems they were having that led to the surgery, and the impact these problems had on their life; what they had hoped and expected from the surgery; whether this was realised; the impact of the surgery on their QoL (positive and negative); and any additional problems or benefits that they experienced following surgery (in the short- and long-term). We focus on the findings relating to these aspects of the interview in this article. We also asked about: their experiences with healthcare professionals and hospital care; their preferences for the format of a new PROM; and any perceived barriers and facilitators to PROM use. This latter focus on facilitators and barriers to PROM use will be reported in a future article. If participants had more than one surgery, we focused on the most recent but with reference to other surgeries if the participant felt this was important. For instance, those who had MC surgery most recently often wanted to discuss their previous M surgery and the impact it had on their QoL, which ultimately led to their decision to have MC surgery.

### 2.5 Analysis

The interviews were transcribed verbatim, and any identifying material was removed. Data collection and analysis were carried out concurrently. Framework analysis (FA) was adopted because “it is flexible, systematic, and rigorous, offering clarity, transparency, an audit trail, and theme-based analysis” (32). In the first stage of FA, JC checked transcripts for accuracy, imported them into NVivo Release 1.6.1. and then began familiarising herself with the data (33). Following this, the data were divided into sections and recurring themes were identified for inclusion in an *a priori* framework.

The initial framework contained broad themes/topics such as clinical problems, outcomes of surgery, pain, psychological/emotional impacts, and role functioning. FA permits the expansion and refinement of existing themes, as well as the incorporation of new themes as analysis progresses and new data is incorporated into the dataset (34). JC coded the transcripts into the framework which was expanded and refined with each interview based on discussions with GLJ. This continued until data saturation, i.e., “the point at which additional data do not lead to any new emergent themes” was reached (35). In this instance, we used code frequency counts, and later theme frequency counts, to establish when no new codes or themes were being generated and when data saturation had been reached. Discussions about the introduction of new themes and sub-themes and potential items for the PROM were ongoing with the team throughout the analysis. FTP later refined the themes for this article further, working from the initial coding framework.

## 3 Findings

### 3.1 Participants

Ninety-five people expressed an interest in taking part in the study and 31 were interviewed. After 20 interviews, gaps in relation to the purposive sampling strategy were considered and efforts made to fill these gaps (e.g., younger people, disabled people, people from a minority ethnic group). Participants’ ages ranged from 35 to 74; the majority were White British with three from ethnic minority backgrounds; area deprivation ratings (acquired using postcode data) ranged from Decile 2 (deprived) to Decile 10 (affluent) with 6/31 in the socially deprived deciles 2–4 (36). Eight participants had other health conditions and/or disabilities including, but not limited to: spina bifida, fibromyalgia, anxiety, depression, migraines and irritable bowel syndrome. Please see Table 1 for further details of all the above.

**Table 1:** Participant information.

ID	Age group	Ethnicity	Area deprivation rating	Other health conditions/disabilities	Occupation	Previous surgery	Condition related to most recent surgery	Most recent surgery	Time since surgery at interview	Outcome	Referral source
01	60–69	WB^a^	6	None	Unemployed (NLW)^a^	None	Anterior/Posterior prolapse	Anterior and posterior repair (POP)	<6 months	Good	Trust A
02	60–69	WB	10	None	Retired	None	Anterior/posterior prolapse	Anterior and posterior repair (POP)	<6 months	Improved	Trust A
03	50–59	WB	6	None	Employed PT	None	Prolapse	Hysterectomy & anterior repair (POP)	2 years–5 years	Good	Trust B
04	70–79	WB	9	None	Employed PT	Mesh for stress incontinence	Anterior/Posterior prolapse	Mesh removal (MC)	6 months–1 year	Improved	Trust B
05	70–79	WB	7	None	Retired	Anterior & Posterior repair	Anterior/Posterior Prolapse	Hysterectomy (POP)	1 year–2 years	Improved	Trust B
08	50–59	WB	9	None	Employed PT	Anterior repair	Anterior/Posterior prolapse	Anterior and posterior repair (POP)	1 year–2 years	Improved	Trust B
12	60–69	WB	9	Complex PTSD; anxiety; vasculitis; autoimmune disease; chronic kidney disease stage 3B	Unemployed (NLW)	Mesh Hysterectomy	Mesh Complications	Mesh removal (MC)	6 months–1 year	Poor	Trust B
13	70–79	WB	10	None	Retired	Anterior repair Hysterectomy	Anterior Prolapse	Anterior repair (POP)	<6 months	Good	Trust C
18	40–49	WB	4	None	Employed FT	None	Urinary Stress Incontinence	Unclear—surgery for stress incontinence (SUI)	1 year–2 years	Mixed—new post-surgery problems	Trust B
23	40–49	WB	9	None	Employed FT	None	Urinary Incontinence	Autologous fascial surgery (SUI)	6 month–1 year	Good	Trust B
24	30–39	WB	8	Degenerative disease in back; fibromyalgia; osteoarthritis; irritable bowel syndrome	Employed PT	None	Prolapse	Prolapse surgery (POP)	6 months–1 year	Good but traumatic experience	Trust C
25	40–49	WB	6	None	Unemployed (NLW)	None	Urinary and bowel incontinence	Birch Colposuspension (SUI)	6 month–1 year	Poor-no improvement	Trust C
26	60–69	WB	9	None	Employed PT	Vaginal repair	Prolapse	Hysterectomy & anterior repair (POP)	6 month–1 year	Good	Trust C
30	50–59	WB	6	None	Retired	None	Urinary Incontinence	Colposuspension (SUI)	6 months–1 year	Good	Trust C
35	40–49	WB	4	None	Employed PT	Trans-vaginal Rectocele repair	Prolapse	Trans-anal rectocele repair and perineorrhaphy (POP)	<6 months	Good	Trust C
36	30–39	WB	3	Long term depression and anxiety	Employed PT	Mesh for urinary incontinence	Prolapse	Anterior and posterior repair for rectocele and cystocele (POP)	1 year–2 years	Poor-worse than before	COM-MASIC (website)
38	30–39	WB	5	Spina bifida; Raynaud's; anxiety & depression	Unemployed (NLW)	None	Urinary Incontinence	Unclear—sling (SUI)	>5 years	Poor	COM-Bladder Health (Instagram)
40	70–79	NWB	10	None	Self Employed	None	Prolapse	Hysterectomy and prolapse repair (POP)	1 year–2 years	Good	Trust A
41	40–49	WB	10	None	Employed PT	TOT Mesh Mesh complications surgery	Mesh Complications	Mesh removal (POP)	2 years–5 years	Poor	COM-STM
43	40–49	WB	10	Hyper-mobility spectrum disorder; Fowler's syndrome	Employed FT	Mesh Mesh complications Mesh removal stage 1	Mesh Complications	Mesh removal stage 2 & birch colposuspension (MC)	>5 years	Good	COM-STM
44	60–69	WB	7	Migraines	Employed PT	None	Urinary Incontinence	Mesh (M)	>5 years	Good	COM-STM
45	30–39	WB	10	None	Unemployed	Sacrohysteropexy mesh	Mesh Complications	Mesh removal & hysterectomy (MC)	<6 months	Improved	COM-STM
51	60–69	WB	8	None	Self Employed	None	Cystocele Prolapse	Anterior colporrhaphy (POP)	6 months–1 year	Improved	COM-STM
54	70–79	WB	2	Mobility problems due to bulging discs	Retired	Mesh	Mesh Complications	Mesh Trimmed (MC)	2 years–5 years	Poor	COM-STM
55	60–69	WB	8	Arteriovenous malformation	Retired	None	Prolapse	Mesh colposuspension & hysterectomy (POP)	>5 years	Initially good but now having problems	COM-STM
60	60–69	WB	4	None	Retired	None	Prolapse	TVTO Mesh (M)	>5 years	Initially good but now having problems	COM-STM
61	50–59	WB	4	None	Employed PT	Mesh for incontinence Fibroid removal	Mesh complications	Mesh removal (MC)	<6 months	Poor	COM-STM
64	60–69	NWB	9	None	Retired	Colposuspension Hysterectomy Mesh	Mesh complications	Mesh removal (MC)	1 year–2 years	Improved	COM-STM
77	60–69	WB	9	None	Employed PT	None	Prolapse	Anterior and posterior repair (POP)	<6 months	Good	Trust A
87	40–49	WB	9	None	Unemployed (NLW)	Anterior and posterior repair	Prolapse	Rectopexy (POP)	>5 years	Poor	COM-Bowel and Bladder (website)
92	50–59	NWB	10	None	Employed FT	None	Prolapse	Hysterectomy & anterior repair (POP)	1 year–2 years	Good	Trust C

a WB, white British; NWB, non-white-British; NLW, not looking for work; PT, part time; FT, full time; COM, community; STM, sling the mesh.

#### 3.1.1 Condition and surgery

The sample included 13 participants whose most recent surgery was non-mesh related surgery for POP, 5 who had non-mesh related surgery for SUI; 13 participants previously had mesh (M) surgery for their prolapse and/or incontinence, 10 of whom had further surgery, 8 described as mesh related complications (MC) and 2 as prolapse surgery (POP). Please see Table 1 for details of the specific surgeries and when these were. Participant ID numbers and abbreviations for the various surgeries are used to label the quotations in the findings section below (e.g., ID24POP is participant 24, surgery for prolapse).

#### 3.1.2 Surgery outcomes

Of the 31 participants, 16 reported having one surgery and 15 more than one surgery. Of the 16 who reported one surgery, 13 had non-mesh related surgery; of these 8 described a good outcome, 3 mixed (improved but still have problems), and 2 (both SUI) reported no improvement or felt their outcome was worse than before surgery. Three participants had surgery where mesh had been used and no further surgery; 1 reported a good outcome, and 2 reported that their outcomes were good initially, but they were having problems at the time of interview. Five participants had repeat non-mesh surgery for prolapse; 3 described a good outcome of the repeat surgery; 1 mixed and 1 poor. The remaining 10 participants described having poor outcomes of mesh surgery before talking about the outcome of their most recent surgery (MC); 2 described being satisfied; 3 described feeling they had mixed/improved and 5 reported having poor overall outcomes following MC.

### 3.2 Themes

In the following sections, we report the nine themes that impact QoL following surgery for POP, SUI and MC. The themes and sub-themes are: short-term impacts of surgery; long-term impacts on pre-surgery symptoms (sub-themes: urine and faecal stress/urge incontinence, prolapse bulge/protrusion, and mesh complications); pain and/or discomfort; impact on daily activities (sub-themes: fatigue, physical activities, caring roles, household responsibilities, and financial resources); social and leisure activities (sub-themes: leisure activities and social life); emotional wellbeing (sub-themes: mental health, embarrassment, anxiety, self and identity, and autonomy); sexual activity; personal relationships; and work/education. These themes have informed the domains and items for the POP, SUI and MC PROM, which is currently being developed. Table 2 shows the themes and sub-themes, with illustrative quotations derived from the data.

**Table 2:** Themes, sub-themes and illustrative quotations.

Theme	Sub-theme	Illustrative quotations
Short-term impacts of surgery	N/A	“I'd had to come home with a catheter on for two weeks, which sort of knocked me sideways to be fair, ‘cause when she'd gone through the wall on my stomach, it had gone straight into my bladder so it burst my bladder. I woke up in recovery room in an absolute agony, wrapped in foil … I expected maybe to have a catheter on when I woke up, but I didn't expect to come home with it and to have it on for nearly three weeks. That, I think that's what psychologically didn't help.” (ID08POP) “I then had this massive lump the size of an orange come up on my vulva … I was sat down, all of a sudden my leg was just covered in blood and it, it was an abscess that had exploded through my scar and all my stitches had had sort of ruptured and this infection had sort of poured out of the scar and it healed. It healed up about three weeks ago. So I've had six months of back and forth to the hospital trying to heal up this hole with numerous numerous infections.” (ID25SUI) “I think it was probably about maybe six, seven months after when things started to feel a bit more normal, but I was just feeling really, really tired all of the time, and I wass till having random aches and like, particularly in one of my thighs, and just being a bit, yeah, just not right *laughs* so yeah, probably like six or seven months after.” (ID23SUI)
Long-term impacts on pre-surgery symptoms	Urine and faecal stress/urge incontinence Prolapse bulge/protrusion Mesh complications	“the pelvic pressure, that disappeared, I could hold my wee for a bit longer, I could have a daily walk, I did my physio exercises, I did generally feel better and I think I was fortunate because I didn’t seem to have any problems, really, I was very lucky.” (ID13POP) “it has messed up my bowels to the extent that I have to go two or three times a day, and sometimes you can be moving about and all of sudden think “Oh my god, I have to get to the toilet”—so really, post op, and prior to the operation, it's affecting my bowels more than the incontinence.” (ID61MC) “I was getting a lot of stomach pains as well, and in June 2,021 I had—this was the start of where I had bowel bleeding, so my bowel was bleeding. It must have bled about 11 times that night and I ended up being admitted into hospital for a week due to the bowel bleeding, then my bowel bleeding kept bleeding practically nearly every other day from June 2021 til the mesh removal.” (ID45M).
Pain and/or discomfort	N/A	“I didn’t have a quality of life because I couldn’t do anything, every time I was in pain, getting constant urine infections. I couldn't stand for more than about three or four minutes because I'd get shooting pains down me legs … I can now stand at the cooker, as long as, you know, I'm not that static.” (ID04MC) “Erm, I've got no, erm, pain *laughs* I'm pain free, I'm not, I'm not having to push my prolapse back in again. Erm, there is no fall out anymore, my, everything has been repaired as well as it can be. Erm, scans post-surgery have revealed that everything has been tightened as well as it can be.” (ID24POP) “As soon as I, as soon as the anaesthetic started wearing off I couldn't sit down and I had a tremendous pull in my back and I didn't sit down for another, until May … had to take a cushion with me everywhere and my rubber ring everywhere when I was in that terrible pain, I just thought I've got to get this out of my body.” (ID64M).
Impact on daily living	Fatigue Physical activities Caring roles Household responsibilities Financial resources	“So, I went back to work pretty much like mid-December, so, and was still feeling really tired at that point and not quite right and then I went back to work after the Christmas break like full on, and just felt tired for quite a long time, a lot of the other, so my wounds had healed fine as far as I was aware, I think it was probably about maybe six, seven months after when things started to feel a bit more normal, but I was just feeling really, really tired all of the time.” (ID23SUI) “I feel great. I feel great. I feel a lot—initially, you've got to get over the surgery etc., but the pelvic pressure, that disappeared, I could hold my wee for a bit longer, I could have a daily walk, I did my physio exercises, I did generally feel better.” (ID13POP) “I can do more with the kids, like we took them to a theme park the other week, you know. Whereas you wouldn't even think about that when I had the mesh. … we used to go for bike rides, this before I got the mesh put in, I can't do any of that still. I want to though, I really want to try, but I know it's too soon. It'll just hurt too much I think. So I can't go bike riding with the kids, so my partner does that with them.” (ID45MC) “I may have been able to do things faster if I'd have allowed myself to, erm, it just took a long time, you know, just to get back, I was very very tired, I was tired all the time, erm, I just, I think I just probably just introduced stuff back into my life, you know like maybe doing a little bit of housework after a few months, erm, but not being silly about things.” (ID30SUI)
Social and leisure activities	Leisure activities Social life	“Before I had the mesh removal, I did have an allotment and I had to give up the allotment due to the pain that I was getting …I still can't go running, you know, I can't go climbing up big hills. I'd love to, but I can't. I used to love hiking and stuff like that, I can't do that. … since I've had the mesh removal I haven't really been able to maintain the garden as such, other than sow some seeds. I did the other day bend down and yeah, try to do a bit, but it's just too much—it just hurts on my back too much. … I can't walk my dog either, because he pulls on the lead and he'll just pull my back. So yeah, there's still things that I can't do.” (ID45MC) “I was gonna say leisure activities like going for a meal, erm, being comfortable enough to sit comfortably, chat, erm, not have to worry about finding a toilet. Erm, yeah, just being able to, I suppose even in terms of being able to enjoy a glass of wine, you know, I can now enjoy a glass of wine, it's brilliant.” (ID24POP)
Emotional wellbeing	Mental health Embarrassment Anxiety Self and identity Autonomy	“I think there's, I think there's a massive improvement in my, in my mental health…there is a financial impact and I haven't got the same life as I had before, in terms of, you know, disposable income, not at all. You know, so I have sacrificed *that*, but I kind of gained other things I suppose, you know, peace of mind and, you know, and just my mental health really.” (ID01POP) “Before the surgery I was so worried, I mean it did happen a couple of times, if the pad became so saturated that it would leak and show through my jeans, so that, it's very embarrassing but now I know that it's not gonna leak, that must, I'm not that aware that it's made me more confident but it must have some sort of impact on my confidence because I know that it's, well I'm pretty sure it's not going to, it's not gonna leak and show through my clothes.” (ID30SUI) “I didn't have a lot of peace about it until I'd got it gone, do you see what I mean? I wasn't worrying constantly about it, but I was aware of it because it's such a physical thing for a woman to have a prolapse.(ID05Pre&PostPr) I think the other thing it did it was impacted on my confidence massively. I don't think I've got that back yet. I think, yeah, it just made me feel, yeah, I just lost confidence in-, in myself … I would like to go back to work cos I enjoyed it, but yeah, I'm, I'm gonna leave it a little while.” (ID01POP) “I do have a little bit of independence, especially more now since the mesh removal than I did before, before I didn't feel like going anywhere or anything like that, whereas now I'm feeling a lot better than I was, so I'm able to drive about here and there, take the kids to visit family and stuff a lot more than what I did before the surgery.” (ID45MC)
Sexual activity	N/A	“The other issue was sex became extremely difficult because it was so painful, and I wanted to have more children.” (ID41M) “I haven't had sex. I feel much better now he's done this perineal repair. … I am nervous about having sex … I mean I'm pretty open with my partner, when, you know, when we've been together, but what would I tell him? Don't do this, don't do that. You know? It's, [sighs] there's huge assumptions that people might know, and I don't wanna tear again.” (ID35POP) “I literally got with my partner about a month, or two months before I had me operation. So, obviously after the operation I weren't allowed to have sex for two months and then when we could it, it were really, I were, I got really upset because I, I cos it didn't, it weren't same it didn't feel same. Just didn't feel right and he were upset because he felt like he, he couldn't satisfy me anymore and it were all because of the operation.” (ID18SUI)
Relationships	N/A	“we seemed to be getting on each other's nerves, if you know what I mean? We, we've been married fifty years, my husband and I. And, yeah, you know, it seemed that I felt he was getting short tempered because he was having so much to do, and he was getting tired. And, I was getting frustrated and annoyed, because I couldn't help him. And I felt like, you know, I felt, well I felt useless. So, it, yes it did affect our relationship.” (ID04M) “I mean things like, I am a single woman, I mean like, the thought of just even going for an initial coffee with someone, you know, I just wouldn't do it, I mean, how embarrassing if it started then. And then what if we get on and we go out for a drink, go on a few dates, and we start having a relationship, there is no way I would let anybody near me sexually at the minute, I just couldn't, you know. It would be mortifying. And how would anyone want to accept that as well, when they don't know you, so yeah, that is a big impact on that area of my life.” (ID12MC)
Work/education	N/A	“[I:You went to flexible working, is that a result of this] Yeah, there was a link to that, I think because of the lifting side of it [my job), I was worried that that could affect me. I think it is quite a heavy job, it can be quite a physical job, so I didn't want it to get any worse. And after the operation I thought is it a good idea to be carrying it on.” (ID03POP) “I've done voluntary work and I don't think I would be able to take on full-time employment, erm, because of it.It depends how you're feeling on the day, I mean I have wet myself at work and I've had to go home. It's not so much the wetting myself, it's the pain and the vomiting, I've gotta take pain, I take cocodomal if I need to, I don't take it as much cos it doesn't really do too much. Erm, concentrating obviously that's quite difficult, so, yeah *laughs*. I don't see much of a future like as I say I've always wanted to work, it's held me back there.” (ID38SUI)

Throughout the findings, we focus on QoL post-surgery, although in places it is necessary to describe pre-surgery experiences to compare outcomes. Table 3 maps the QoL themes against the patients for whom there was an improvement post-surgery, and patients for whom it remained a burden, worsened, or new issues developed post-surgery. Table 3 also includes descriptive statistics to demonstrate how data saturation was monitored and to highlight the range of positive and negative outcomes reported by participants. Where no condition/surgery is specified, the data is representative of all three patient groups (POP, SUI and MC) such that at least one participant from each patient group reported a problem related to that theme post-surgery. Where there is an identified difference between the patient groups, this is reported.

**Table 3:** Data saturation.

Theme	Patients for whom there was an improvement	Total	Patients for whom this worsened or remained a burden, or new symptoms developed	Total
Symptoms (short and long-term)	SUI* *n* = 3 *(IDs 18, 23, 30)* POP* *n* = 7 *(IDs 02, 03, 13, 35, 40, 77, 92)* MC* *n* = 4 *(IDs 41, 43, 45, 54)*	14 (45%)	SUI *n* = 4 *(IDs 18, 23, 25, 38)* POP *n* = 6 *(IDs 02, 08, 24, 35, 40)* M* *n* = 3 *(IDs 44, 55, 60)* MC *n* = 6 *(IDs 04, 12, 41, 45, 61, 64)*	19 (61%)
Pain and/or discomfort	SUI *n* = 1 *(ID 25)* POP *n* = 7 *(IDs 02, 03, 13, 24, 26, 40, 92)* MC *n* = 6 *(IDs 04, 12, 41, 43, 45, 64)*	14 (45%)	SUI *n* = 3 *(IDs 23, 25, 38)* POP *n* = 3 *(IDs 08, 36, 35)* M *n* = 3 *(IDs 44, 55, 60)* MC *n* = 3 *(IDs 41, 54, 61)*	12 (39%)
Impact on daily living	SUI *n* = 4 *(IDs 18, 23, 30, 38)* POP *n* = 8 *(IDs 01, 02, 03, 05, 13, 24, 35, 36)* M *n* = 1 *(ID 44)* MC *n* = 3 *(IDs 04, 43, 45)*	16 (52%)	SUI *n* = 2 *(IDs25 38)* POP *n* = 2 *(IDs 35, 51)* M *n* = 2 *(IDs 55, 60)* MC *n* = 3 *(IDs 41, 61, 64)*	10 (32%)
Social and leisure	SUI *n* = 2 *(IDs 23, 30)* POP *n* = 4 *(IDs 03, 05, 13, 24)* MC *n* = 3 *(IDs 04, 45, 54)*	9 (29%)	SUI *n* = 2 *(IDs 25, 38)* POP *n* = 2 *(IDs 01, 36)* M *n* = 2 *(IDs 55, 60)* MC *n* = 5 *(IDs 12, 41, 54, 61, 64)*	11 (35%)
Emotional wellbeing	SUI *n* = 2 *(IDs 23, 30)* POP *n* = 10 *(IDs 01, 03, 05, 13, 24, 26, 35, 40, 77, 92)* M *n* = 1 *(ID 44)* MC *n* = 4 *(IDs 04, 43, 45, 54)*	17 (55%)	SUI *n* = 2 *(IDs 18, 38)* POP *n* = 5 *(IDs 08, 25, 35, 36, 54)* M *n* = 3 *(IDs 44, 55, 60)* MC *n* = 4 *(IDs 12, 41, 61, 64)*	14 (45%)
Sexual activity	SUI *n* = 1 *(ID 30)* POP *n* = 3 *(IDs 08, 24, 35)* MC *n* = 1 *(ID 45)*	5 (16%)	SUI *n* = 2 *(IDs 18, 25)* POP *n* = 2 *(IDs 36, 51)* M *n* = 3 *(IDs 44, 55, 60)* MC *n* = 3 *(IDs 12, 41, 64)*	10 (32%)
Relationships	POP *n* = 1 *(ID 08)* MC *n* = 1 *(ID 45)*	2 (6%)	SUI *n* = 1 *(ID 38)* POP *n* = 1 *(ID 35)* MC *n* = 1 *(ID 12)*	3 (10%)
Work/education	POP *n* = 3 *(IDs 24, 35, 40)* MC *n* = 2 *(IDs 04, 43)*	5 (16%)	SUI *n* = 3 *(IDs 18, 23, 38)* POP *n* = 2 *(IDs 24, 36)* M *n* = 2 *(IDs 55, 60)* MC *n* = 3 *(IDs 41, 61, 64)*	10 (32%)

#### 3.2.1 Short-term impacts of surgery

Most participants described their recovery from surgery as difficult, but they had expected this. Participants experienced short-term increases in pain, fatigue and incapacitation which are associated with any major surgery:

“But it were [sic] quite severe for a week, I would say after surgery. Like it, I struggled to even walk up the stairs because you don’t realise how much you use your stomach muscles to just walk up the stairs […] I don’t get anything now, it’s fine [it was] the nature of the surgery.” (ID04M)

Some participants suffered complications (e.g., post-operative infections, urinary tract infections, allergic reactions to medication, blood loss, passing of blood clots, burst bladder, hernia resulting in bowel blockage, constipation, and urine retention) immediately after surgery which impacted quality of life and led to the occurrence of new symptoms:

“I then had this massive lump the size of an orange come up on my vulva […] it was an abscess that had exploded through my scar and all my stitches had sort of ruptured and this infection had sort of poured out of the scar [..] So I've had six months of back and forth to the hospital trying to heal up this hole with numerous infections.” (ID25PostSUI)

Most of these complications were relatively short lived, although recovery could be longer than expected, many reporting 6 months or more:

“It took me I’d say probably more like almost 12 months to actually be able to start doing a bit more without having to lay down or sit down or go to bed etc.” (ID24POP)

The most common problem reported in the short-term post-surgery was the need for catheterisation, including the shock and distress that a catheter was required, difficulties changing it, leaking, infection and associated pain:

“I ended up covering myself in wee about, well, for the first few days I was just crying, crying because I just didn’t know what to do every time this thing leaked or whatever.” (ID01POP)

#### 3.2.2 Long-term impacts on pre-surgery symptoms

Before discussing the impact of surgery on quality of life, we first outline what the participants told us about how the surgery impacted (or did not impact) their pre-surgical conditions and symptoms. Many participants reported improvement in their symptoms and conditions post-surgery, but some reported that they persisted, worsened, or that they developed new symptoms. We outline these in more detail below.

##### 3.2.2.1 Urine and faecal stress/urge incontinence

Pre-surgery, a large proportion of the participants reported bladder and bowel-related problems including stress incontinence and/or urge incontinence (a sudden and intense need to urinate or defecate and difficulty holding it in). Also reported were problems with urine retention, where the bladder did not empty sufficiently, often due to a prolapse pressing on the urethra. This could result in considerable discomfort and recurrent urinary tract infections. Some participants also reported bowel problems, particularly those who had a rectocele (prolapse of the rectum into the vagina). Bowel symptoms included fecal incontinence, bowel urgency, and symptoms of irritable bowel syndrome including bloating, excessive gas, loose stools, mucus and constipation. Both bowel and bladder problems resulted in the need for frequent visits to the toilet and/or to be near a toilet. Many participants had good outcomes from surgery and reported that their incontinence had improved or stopped completely:

“What I was hoping for from that surgery was that I had absolutely no leaks ever again. That was my absolute perfect outcome would be that. [I: Okay and has that been the case?] It has, hurrah!” (ID30SUI)

Others reported that although their stress incontinence had improved, they had now developed urge incontinence post-surgery:

“I was literally, just involuntarily poo was coming out of me…this is happening 3 or 4 times a day. But what I’ve been left with now is, suddenly I have an urge and if I know it’s not a firm poo, if I don’t make it, I will leak before I get to the toilet. And it’s like, I didn’t have that before [the surgery].” (ID36POP)

For others, their incontinence had improved or stopped following surgery, but other, new symptoms had developed:

“I see from my notes that my doctor was highly delighted because I wasn’t incontinent anymore. But I paid the price because I was having infections and discomfort all the time … I wish I hadn’t had it done. I wish I’d just put up with the incontinence.” (ID54M)

##### 3.2.2.2 Prolapse bulge/protrusion

Pre-surgery, those who underwent surgery for prolapse reported experiencing an uncomfortable bulge or lump which protruded out of the vagina. This could be associated with urinary incontinence and retention, as well as feelings of shame and embarrassment. Many participants who had prolapse repair surgery had good outcomes, and they reported that the bulge/protrusion was gone following surgery:

“I’m not having to push my prolapse back in again. There is no fall out anymore, everything has been repaired as well as it can be.” (ID24POP)

Others reported that they were happy that they no longer had a protrusion, but that other symptoms persisted, such as urge incontinence:

“After surgery is fantastic […] I feel completely normal […] If my brain decided that I have to go to the toilet I have to go immediately […] There is no prolapse anymore. Everything is fixed […] Before I had to have the toilets to go and try to put my prolapse inside, now I only have to think if there will be a toilet on my way like most of the older woman have this problem [sic]” (ID92POP).

##### 3.2.2.3 Mesh complications

It is important to highlight that most participants who underwent mesh removal surgery experienced some improvements in their symptoms and/or pain but reported that other symptoms persisted. They did not believe that the symptoms that persisted were caused by the mesh removal surgery, rather, they felt that the mesh that was initially implanted had done irreparable damage to their bodies:

“I know that I have had a foreign body response to the mesh [.] Because the pins were basically wedged in my sacrum […] she tried to remove them, the surgeon, but it just wasn’t safe to remove […] it still feels like it’s snapping when I bend down and it still burns and I still get the tingles running down my legs, but out of 10 I’d say it’s about a 4 out of 10 now, it’s a lot better […] So it’s definitely improved and the bleeding from my bowel has improved as well.” (ID45MC)

#### 3.2.3 Pain and/or discomfort

Pain or discomfort was the most reported symptom that impacted QoL. Most participants described some pain or discomfort regardless of their condition. Prior to surgery, participants tended to describe discomfort including: ‘*period-like stomach pains’*; ‘*dragging, aching pain’*; ‘*a bulging feeling’*; ‘*feeling sick’*; the feeling of a need to go to the toilet, and feelings of ‘*pressure’* in the pelvic area. For those in the sample who had discomfort or pain caused by prolapse, their surgeries usually improved their symptoms:

“Yeah, the prolapse, he’s definitely sorted that out and I’m not in the pain that I was, because I started to get a really bad dragging pain down my side.” (ID02POP)

Pain and/or discomfort were commonly reported post-surgery, both in the short and long term. In the short term, some participants reported severe pain immediately after surgery which took longer than they expected to subside. This was associated with post-surgery infections, urine retention, catheter pain or pain from an unknown source. Significant and debilitating pain was experienced by those who had mesh surgery. For some, the pain started immediately after surgery and did not improve until after corrective surgery had been performed:

“Instantly on waking up I was in agony […] I was in constant pain in my back […] there were days where you just couldn’t do anything—you just try and bend down and pick up something off the floor, like a dog toy, you just couldn’t do it because your back’s constantly feeling like it’s snapping and burning.” (ID45M)

For others the pain started some years post-surgery. This ‘*agonising*’ or ‘*debilitating*’ pain could be in the back, hips, down the legs, in the pubic, pelvic and stomach areas or generalized all over the body. The pain was variously described as: ‘*like a cheese-wire or a knife going through me’*; ’*shooting pains’*; ‘*cramping*’, ‘*a pulling in the back*’ or ‘*burning*’ or ‘*tingling*’ feelings which they associated with nerve damage from the mesh surgery:

“I couldn’t stand for more than about three or four minutes because I’d get shooting pains up me, err, down me legs [sic].” (ID04MC)

Participants underwent MC to deal specifically with the pain they were experiencing, which they believed was a direct result of the mesh being implanted. Thankfully, following mesh complication/removal surgery, the pain had gone completely or had significantly improved for several participants, whilst some described being left with ‘*residual pain’* or ‘*occasional twinges’*:

“Most of the pain has gone, I have got a slight, what I would call, not pain like it was, I have got sore-, a bit of soreness in one area, and a little bit under the scar at one end. Erm, but the pain from walking, and in the hip and the knife pain, that has gone.” (ID12MC)

#### 3.2.4 Impact on daily living

The participants described the impacts that their surgery had on daily living, including their ability to undertake physical activities, caring and household responsibilities, and financial resources.

##### 3.2.4.1 Fatigue

Post-operative fatigue was commonly reported. This was mostly short term but for some it lasted longer than they expected. Participants associated this with the anaesthetic, post-operative pain, urinary infections, or disturbed sleep due to pain, incontinence or worry. Fatigue had the greatest impact where there was a necessity to return to work. Chronic fatigue, which occurred long after surgery, was reported mostly by participants post mesh surgery. It was described as ‘*beyond tiredness’* together with feeling generally unwell, something some participants associated with auto-immune disease. ‘Brain fog’ and difficulties concentrating were often reported alongside fatigue:

“I started getting awful fatigue which is like your autoimmune system upset, when you’ve got that awful fatigue, it just totally changes your personality and everything is a struggle to complete. You’re sort of in that fog.” (ID61M)

In contrast, for some participants, surgery resulted in increased energy levels in the long term:

“I felt so much better for having it done—younger even. I mean I am now 72 and I feel I’ve got more energy.” (ID05POP)

##### 3.2.4.2 Physical activities

After a period of recovery, many participants were able to resume the physical activities that became hard or impossible to do with their pre-surgery condition/symptoms:

“Now I feel great. I mean, now I can walk in the countryside all day if I want to. I feel very comfortable. I do some yoga. In fact, before, I’d be doing yoga and that would feel a bit uncomfortable whereas now I can do that and I feel comfortable doing it. Yeah, it feels brilliant, just feels so normal”. (ID03POP)

Other participants described difficulty engaging in physical activity post-surgery, such as walking, running, swimming, cycling, and general exercise. Many felt that they had had to abandon a healthy, active lifestyle which would aid their overall well-being, particularly post mesh surgery:

“I used to go to the gym a couple of times a week, and I had to give that up…it’s my own general fitness, I need to get that back.” (ID04MC)

After successful surgery, getting back to physical exercise could take longer than hoped for some, and was done with caution:

“I’m not doing exercise at the moment which is frustrating cos I’m putting weight on, because I don’t know whether I can really do exercise or not.” (ID35POP).

##### 3.2.4.3 Caring roles

Many of the participants had caring roles for children, partners or elderly parents. Sometimes, the participants’ conditions and symptoms meant that they struggled to perform these roles pre-surgery. Those who reported successful surgery spoke of how, after a period of recovery, they were able to resume their caring activities:

“It’s a dream. Today we went to soft play, we spent all morning in a soft play area going up and down cargo kind of nets and going down slides and climbing and me lifting, you know, me supporting him and pushing him over things and I don’t even think about it now. It’s just, you know, yeah, it’s great.” (ID24POP)

In the short-term period post-surgery, many participants reported that it had been difficult to perform their caring roles due to pain, physical limitations (particularly lifting), fatigue and generally feeling unwell. Participants described the difficulties of performing the normal role of being a mother, or grandmother, and being involved with normal child play and caring activities. This could negatively impact on children who sometimes had to become the carers:

“I was just laid on the sofa, basically crying in pain and my poor kids had to basically help support me. They’ve had to basically grow up a bit too quick.” (ID45M).

##### 3.2.4.4 Household responsibilities

Participants described difficulties performing daily household chores due to physical limitations, pain and fatigue. This was particularly evident immediately post-surgery where lifting anything of any weight caused difficulties with shopping, cleaning, washing and cooking:

“I’d been told, you know, that I can’t lift anything. And, that’s easily said, you know, because you don’t think, you don’t realise how much you are lifting in your normal everyday life.” (ID51POP)

Long term pain, and the subsequent physical limitations, particularly post mesh surgery, could also make carrying out day to day household activities difficult or impossible. Some participants spoke of being reliant on other people for support:

“… and then the lifelong of needing cleaners, gardeners, needing care in the house to support you to do stuff.” (ID41M&MC)

##### 3.2.4.5 Financial resources

For some participants who were unable to work post-surgery (see Section 3.2.9), there was a direct impact on their financial resources meaning that they had to rely on disability benefits, find money from other sources (savings, re-mortgaging, down-sizing, family, fund-raising, compensation) or become dependent on a partner’s income:

“My care alone just getting a nanny to support three years of my children cost £45,000, money we didn’t have. We had to re-mortgage our home, it’s just ridiculous. So, and then loss of earnings, 3 years I couldn’t work.” (ID41M)

Loss of earnings also meant that they no longer had the finances to do the things that would improve their quality of life, such as social events and holidays:

“there is a financial impact and I haven’t got the same life as I had before, in terms of disposable income, not at all.” (ID01POP).

There were also associated costs post-surgery that put a drain on resources, the largest being the necessity for some to access private medical care following mesh surgery, and childcare. Other costs included sanitary ware, incontinence pads, dressings, replacement clothing, prescriptions, housekeeping, and self-help programmes. For those whose symptoms improved, they also had access to more funds, recouped from, for example, not having to wear pads anymore:

“just not having to constantly wearing pads, so that’s been a massive improvement.” (ID23SUI)

#### 3.2.5 Social and leisure activities

The participants also described the impacts that their surgery had on their ability to engage in social and leisure activities which improved their quality of life.

##### 3.2.5.1 Leisure activities

The physical limitations associated with their symptoms and pain pre-surgery made it difficult for participants to do the activities they found enjoyable or made those activities less enjoyable. This included leisure activities such as going on holidays, days out, walking in the countryside, walking the dog, sporting activities and hobbies. For many, they were able to resume the activities they enjoyed following a period of recovery post-surgery:

“there’s like a walking group we wanted to join but I thought, “No, I’m not doing it” because I thought I don’t really want to 1) wet myself, or be like-, be just thinking about that the whole time. … Once I decided to go for it, the surgery, it’s just like yeah—so now it is just get back into enjoying life again!” (ID02POP)

“I haven’t been fishing yet, but I think I will be able to because I’ve no pain to stop me doing the things now.” (ID04MC)

For other participants, particularly those who had mesh complications, their surgeries had greatly impacted their ability to partake in leisure activities:

“I have got three like bucket list holidays booked, and I can’t do any of them, and I asked the surgeon on Thursday, the consultant, and asked ‘am I fit enough to go abroad yet’ and he went ‘No’ […] I really could do with just like lying on the beach, feeling the sand and the sea and having a paddle and the sun, and like, you know, the smells of being abroad, I miss that! You know, just not being able to do what you really feel will pick you up, that’s quite difficult.” (ID03POP)

##### 3.2.5.2 Social life

For some participants, having the surgery had meant that they could resume the activities (e.g., exercise, sport, leisure activities and hobbies) that were intertwined with their social lives because their symptoms/conditions had improved:

“I have been able to do other activities without worrying about it, like paddle-boarding and meeting up with friends and just not having to constantly wear pads, so that’s been a massive improvement.” (ID23SUI)

“Well, that means I can now get out, I can go to the coast, or I can go and see friends, I can get to see family, I haven’t got the fear of what might happen, I just feel like a free agent.” (ID13POP)

For others, having the mesh implanted and waiting for mesh removal surgery meant that they felt their lives were on hold, including their social lives:

“You just lose friends, not because they want to but if you stop going out to do those activities, you know, life moves on for everybody, so you become more isolated, more lonely.” (ID12MC)

Some participants experienced problems using public transport or driving to partake in leisure or social activities due to the potential for embarrassment if they leaked, because of pain, or due to their concerns about ‘undoing’ surgery, particularly in the short-term post-surgery. This also affected feelings of autonomy and independence (also see the next theme on emotional wellbeing):

“I don’t think I drove for about 3 months because I was, I was just worried about undoing the surgery… it made me feel a bit dependant on people” (ID30SUI)

#### 3.2.6 Emotional wellbeing

The experience of needing and having surgery, and the impact this had on their everyday lives, affected participants’ psychological well-being and mental health. This included how they felt about themselves, their confidence and self-esteem and their sense of autonomy and independence. Where surgery was successful, emotional well-being was described as improved.

##### 3.2.6.1 Mental health

Prior to prolapse and incontinence surgery participants spoke of feeling ’*sad*’, ‘*miserable*’, ‘*upset*’ ‘*demoralised*’ which was usually the point at which they sought help. Many found that their mental health improved post-surgery and spoke with a sense of optimism:

“it felt very negative before and now I feel more positive. I feel better psychologically, definitely.” (ID03POP)

Some participants reported a negative impact on their mental health post-surgery. This could be relatively short lived due to immediate post-surgery complications, although was sometimes affected by recovery taking longer than expected:

“I just wondered if it was because I’m older. I don’t know. I don’t know, just (pause) I didn’t realise it would take so long to get your mojo back. Your energy and your interest back into things.” (ID02POP)

For those with long-term problems post-surgery, particularly after mesh surgery, the impact on their mental health led some participants to seek professional psychological help due to feelings of depression, severe anxiety, trauma and post-traumatic stress disorder. For a small minority of participants, the whole experience left them with suicidal thoughts:

“Sometimes I just felt I didn’t want to be here anymore”. (ID04M)

##### 3.2.6.2 Embarrassment

For participants whose surgery had resulted in the development of incontinence, they reported feelings of humiliation, embarrassment or shame associated with public exposure because of leakage, potential smell due to leakage or infections, and exposure due to repeated trips to the toilet:

“… you know, in your forties and you wet yourself all the time is quite degrading, humiliating.” (ID25SUI)

For participants whose incontinence symptoms improved following surgery, they reported feeling less embarrassed and worried about leaking, which had a positive impact on their QoL and ability to engage in activities:

“I have not leaked, so I am not having to wear pads, I am able to walk […] and not worrying about leaking, and the embarrassment of that.” (ID23SUI)

##### 3.2.6.3 Anxiety

Participants reported a fear of making their condition worse, or undoing the benefits of surgery, particularly immediately post-surgery. Consequently, they adopted a ‘better safe than sorry’ attitude which meant that activities were carried out with caution, or not at all:

“I was just worried that the surgery was all gonna come undone and I’d be back to where we started, erm, so it might have just been me being a bit overcautious, so maybe that slowed my recovery down, I may have been able to do things faster if I’d have allowed myself to.” (ID30SUI)

Other participants, particularly those who had mesh complication surgery and thought that some mesh remained in their body, explained that they lived in fear of something else happening:

“I do worry about what the surgery has done to the sling, what’s going to happen for the future, you know, what’s ahead of me, whereas I’ve never thought about that before, but that’s only recently. Because now I worry did the surgery move it, you know, and when I’m feeling pain in my abdomen, which is probably from the surgery, I’m thinking, is that the mesh? What’s happening?” (ID44M)

##### 3.2.6.4 Self and identity

Some participants expressed feeling a loss of self-worth, useless, and losing a sense of who they once were because of their symptoms and conditions, which remained a burden post-surgery:

“you are not a person who likes dancing, likes going for walks, likes doing this, likes meeting up, likes going for a coffee, likes going for a drink with her friends, you are not that person anymore.” (ID12MC)

Some also spoke of losing their sense of ‘*womanliness*’ because of problems around their sex lives: not feeling attractive, not being able to wear the clothes that reflected their personality and putting on weight due to lack of exercise. There was a feeling of being ‘*abnormal’, ‘flawed’* and *‘like a freak’*. For some, surgery helped them to feel ‘*normal’* again:

“it feels brilliant, just feels so normal. I actually-, I probably felt abnormal before. Now I just feel more normal.” (ID03POP).

##### 3.2.6.5 Autonomy

Some participants spoke about losing their independence and having to rely upon others to help them, particularly in the short-term period after surgery, and for those who had mesh surgery:

“I wasn’t, I was no longer able to do things for myself, and I’ve always been virtually, you know, really independent. And, I felt like I was ninety and everybody was having to do things for me.” (ID04M)

#### 3.2.7 Sexual activity

Limitations to sexual activity were frequently reported, particularly by younger participants and those whose sex lives were important to them. Pain was described as impacting on sexual activity following surgery, in both the short and long term. This resulted in physical inability or difficulty having sex, or an avoidance or disinclination towards an active sex life as it was no longer enjoyable:

“It wasn’t a good experience of the pelvic floor repair […] my experience of that was that it wasn’t done very well because several months afterwards, when my husband and I attempted intercourse, I was in excruciating pain.” (ID05POP)

Other sexual repercussions post-surgery included a lack of sensation in the vagina, affecting the ability to orgasm, and alterations to the vaginal opening, which meant that penetrative sex was difficult or impossible:

“he'd sewn me up completely, I hardly had any opening in my vagina… I couldn't have sex in all that time because there was no opening.” (ID64M)

Some participants spoke about the ways they had adapted to sex with their partners following surgery by, for instance, using toys and creams:

“you know, we have adapted and used toys. So, that, that’s made things a bit better and that’s what we have to do now […] But that were [sic] me biggest issue from having surgery.” (ID18SUI)

For others, particularly those with incontinence which led to embarrassment, surgery had greatly improved their symptoms, self-identity and as a result, sexual activity:

“I’d feel unclean all the time […] it kind of just affected me […] Sex life is much better because we, you know, get back to where we were on that one, erm, just the whole general thing is, is great, is perfect.” (ID30SUI)

#### 3.2.8 Intimate partner relationships

One of the greatest impacts reported on partner relationships was the reluctance or inability to engage in sexual activity due to pain or embarrassment (described earlier). Participants reported that this resulted in a loss of intimacy and could create a distance between partners which could further put a strain on the relationship. There were also concerns about the impact this was having on partners and resulting feelings of guilt:

“I didn’t want to have sex and then he thought that he was the problem when it wasn’t and I was just embarrassed about it all.” (ID18SUI)

The consequences of ill health could also put a strain on partner relationships by increasing dependency, with the partner having to undertake roles previously undertaken by the participant, or the partner having to vicariously deal with the stress and mental health consequences of the condition or surgery:

“I’ve ended up sort of separated from my partner. It’s quite early. That was not helped by the stress that I was under immediately after surgery. He has his own difficulties and he struggled to manage that stress.” (ID35POP)

Not only were current relationships impacted, but also the potential of having an intimate relationship with someone in the future, which participants found upsetting:

(I: “Would it be impacting on future relationships?”) “Yeah I think it would, yeah. Erm, sorry that’s the difficult part [cries], erm, because I want a life, but I don’t see it possible. No. Sorry [cries].” (ID38SUI)

#### 3.2.9 Work/education

Those who were in paid work or education described how pain, fatigue, infections, ‘brain fog’, physical demands and limitations (particularly lifting), incontinence, or feeling generally unwell impacted their ability to work and study. This could be at any stage, but particularly immediately post-surgery and longer term for those who had mesh surgery. For others, surgery improved their ability to work or study:

“I found that I couldn’t sit at the computer for long. I’d sit half an hour maybe and do some work and then I’d have to change positions or, you know, just leave it for that day… Now I’m, I’m fine. I work, well, I work on me [sic] laptop […] I take my laptop wherever I go now.” (ID04MC)

Some had to leave their jobs entirely, and have never worked since, whilst others reduced their hours or changed the type of role they performed, which had significant financial consequences (see earlier). Participants also reported taking long periods of time off work, particularly after surgery, which could either put their jobs in jeopardy, or caused anxiety as they feared losing their jobs:

“I had to take time off work for my surgery and my recovery, but then because I had recurrent UTIs I just wasn’t well and I ended up having to have more time off to recover […] I had to have an absence review because of my time off after surgery.” (ID23SUI).

Out of financial necessity, or because they enjoyed their work, some continued to work, often with difficulty. Participants reported making adaptations within their workplaces if they could:

“If I wasn't able to carry out my job then they would get rid of me […] I'd have perching stools and I wouldn't have to go upstairs, and I'd make sure that there was a toilet close by.” (ID64MC)

As well as current employment, future careers were also affected, and some participants had to resign themselves to not progressing within their careers, particularly after mesh surgery:

“so now I live with a prolapsed bowel, completely prolapsed bladder, prolapsed vagina, and urgency that’s really bad, to the point where if I have to work away from the house I will wet myself constantly… I work from home now… I had a career, I had aspirations to be a [senior manager] and do all kinds of things, but now I can’t.” (ID41MC)

## 4 Discussion

### 4.1 Summary of findings

This study aimed to identify all aspects of QoL that may be impacted by surgery for POP, SUI and MC, to inform the development of a new PROM. We identified nine themes for potential inclusion in the PROM, indicating that the potential impact of pelvic organ surgery for SUI, POP or MC on quality of life is multidimensional and complex.

The impact of surgery on the thematic areas of QoL which were identified could be positive, negative, or mixed. Those participants whose surgery was successful mostly described an improvement in many of the QoL themes. For some participants, post-surgery complications and recovery had a more negative impact on the QoL themes than they expected, but their outcomes were generally positive in the long term. However, some participants described many aspects of their QoL as being worse than before surgery, primarily, but not confined to, those who had some form of mesh surgery. It is important to note however, that those who had MC surgery did describe some improvements (e.g., pain) and attributed their poor overall QoL to their previous mesh surgery and the negative impacts this continued to have, even following corrective MC surgery.

### 4.2 Contributions to the literature

Our research resonates strongly with other qualitative studies that have been undertaken with patients having surgery for POP, SUI and MC. These studies identified the impact of adverse events immediately post-surgery (23) and the functional effect of symptoms longer term on multiple life domains including psychologically, socially, sexually, and physically (18, 23, 24). Most of these studies focused on one type of surgery only, particularly mesh surgery (15–19, 21). Our study complements these existing studies by highlighting themes that resonate across a range of conditions and surgeries.

To improve quality of care, it is necessary to examine and understand the relationship between dimensions of health-related quality of life (HRQoL) (37). When compared to the most widely cited model of HRQoL developed by Wilson and Cleary (38), we can see that our findings support their assertions of a relationship between condition and associated symptoms, functioning, health perception and QoL in both a positive and negative way. Likewise, we show the dominant pathway from symptoms, through functioning, to perceptions of wellbeing. Our analysis also revealed evidence of multi-directional associations. We found that as well as symptoms (e.g., pain) impacting on functioning, the inability to function and the losses associated with this (e.g., social, leisure, roles) impacted psychological well-being, which further impacted on functioning and overall QoL, which may or may not be improved with surgery.

The impact of symptoms and limited functioning on the participant's concept of self (i.e., their identity, feeling abnormal, loss of confidence and self-esteem) was not explored in Wilson and Cleary's model but was indicated in Ferrans et al's (39) updated model, which explored the impact of both individual and social or environmental characteristics on domains. Our study adds to the Wilson and Cleary model by showing that a person's ability to perform a task can be affected by psychological as well as physical barriers. Shame, embarrassment (i.e., how they feel they will be perceived by others) and anxiety meant that participants in our study were ‘able’ to perform a task but often chose not to because of psychological barriers.

Of note is the potential differential impact of functional losses dependent upon a person's age, personal circumstances and their values and beliefs. Our analysis indicated there may be a greater impact of the condition, and outcome of surgery, on young women as they could be wanting to extend their family, be looking after young children, be at the start of their careers, and be more inclined towards vigorous exercise, which they have had to give up. Being unable to partake in sexual activity could be devastating for some, and inconsequential for others. We found that older participants seemed more likely to accept their condition/symptoms as a ‘natural part of aging’ and would have lower expectations regarding their functional abilities. This could be explained by the importance of a person's values and expectations, and any PROM would need to be able to address this differential (36).

### 4.3 Strengths and limitations

It was our intention to recruit a diverse sample to the study. We succeeded in recruiting patients from different sources, of different ages, from diverse social backgrounds, with disabilities/co-morbidities, and who had a range of conditions and surgeries. However, despite our efforts, we recruited few women from ethnic minority communities. This is a shame because, as is widely reported, ethnically minoritised women are more likely to experience health inequality across their life course and continue to be underrepresented in health research (40, 41).

It is important to note that self-selection bias (i.e., people choosing to take part *because* they had specific experiences and wanted to tell their story) may have played some part in recruitment for this study. For instance, participants who were recruited through community organisations mostly reported problems post-surgery and subsequently, there may be an over-emphasis on the negative aspects of QoL in our findings. However, self-selection bias may also be true of those recruited through the NHS hospitals, and indeed anyone who chooses to take part in any research (both qualitative and quantitative) of their own volition, as per ethical requirements (42). We consider it a benefit of the research that the full spectrum of experiences is represented, particularly because part of the rationale for developing a new PROM was to ensure that adverse outcomes could be identified.

## 5 Implications

It is important that PROMs are grounded in the experiences of patients (43). Our findings highlight the aspects of QoL, from the perspective of patients, that were impacted by surgical interventions for POP, SUI, and MC. The themes we outline in this paper will inform the various domains and items of the PROM. Thus far, these qualitative findings have been combined with evidence from systematic reviews (28, 29), secondary analysis of existing data (31), and stakeholder involvement to identify a long list of items for testing in a new PROM. This mapping exercise will produce the conceptual framework for the PROM, and we will report the specifics of how the themes were translated into domains and items in a future publication. The new PROM will be available for use within health care settings and research studies soon.

## 6 Conclusions

The impact of surgery for POP, SUI and MC on QoL is multidimensional and complex. The PROM that is under development for these surgeries needs to include the potential for improvement and/or eradication of symptoms, the failure of surgery to improve symptoms, and the development of new symptoms after surgery. The PROM should also reflect the consequential positive, negative and mixed impacts that POP, SUI and MC surgery can have on various aspects of QoL, including pain, daily living, social and leisure activities, emotional wellbeing, sexual activity, relationships and work/education, in both the short- and long-term.

## Data Availability

The original contributions presented in the study are included in the article/Supplementary Material, further inquiries can be directed to the corresponding author.
